# Supplementary material for: Are carbon emissions trading and green financial instruments synergistic? -Comprehensive quantitative research based on content analysis
Source: PLoS One. 2024 Mar 7;19(3):e0298601. doi: 10.1371/journal.pone.0298601 (PMC10919601; doi:10.1371/journal.pone.0298601)
Supplement: S1 Data — (ZIP) [file pone.0298601.s001.zip › Data/地级市Z_F工作报告.zip!/╔╜╬≈/╦╖╓▌╩╨/└┤╫╘╣1⁄2╓┌║┼í╛┬φ┐╦╩2╛▌═°í┐_2012.docx]

各位代表：

　　现在，我代表市人民政府向大会作工作报告，请予审议，并请市政协委员和其他列席人员提出意见。

　　一、2011年工作回顾

　　过去的一年，在省委、省政府和市委的坚强领导下，我们深入贯彻落实科学发展观，大力弘扬右玉精神，坚持以科学发展为主题，以加快转变经济发展方式为主线，坚定不移推进转型跨越发展，全力打造新基地新优势新朔州，全市经济社会保持平稳较快、健康协调的发展态势，实现了“十二五”时期良好开局。

　　(一)经济发展再创新业绩。在连续几年高位运行的基础上，经济增长速度进一步提高。全市地区生产总值完成855．2亿元，增长15．2％；财政总收入完成175亿元，增长32．1％；一般预算收入完成70．9亿元，增长27．2％；工业增加值完成472亿元，增长22．3％；固定资产投资完成474．6亿元，增长33．3％；社会消费品零售总额完成l64．1亿元，增长17．6％；城镇居民人均可支配收入达到20287元，增长15．5％；农民人均纯收入达到7024元，增长19％。主要经济指标绝对额在全省位次普遍前移，多项指标增幅居全省前列。

　　(二)转型发展实现新突破。煤炭资源整合煤矿兼并重组圆满完成。经过连续几年的攻坚克难，我市在全省率先完成煤矿兼并重组，我们彻底告别了小煤窑时代，进入了现代化大矿时期。整合重组效果进一步显现，去年全市煤炭产量达到l．86亿吨，位居全省第一、全国前列。电力工业进一步壮大。右玉京玉2×30 万千瓦、金海洋昱光2×30万千瓦、同煤怀仁2×20万千瓦3座煤矸石电厂和8座风电场、2座太阳能光伏电站投产运行，应县晶都太阳能二期和应县生物质能电厂等新能源项目开工建设。全市电力装机容量达到604万千瓦，能源工业基地地位进一步巩固。循环经济发展取得重要进展。固废综合利用工业园区基础设施建设基本完成，北京大学工业固废综合利用产学研示范基地和粉煤灰提取氧化铝白炭黑等项目开工建设，工业固废资源再生产业基地建设走在全国前列。现代服务业加快发展。居然之家建成运营，现代物流业加快扩张。玉龙生态园等26个旅游项目开发建设，旅游业呈现出蓬勃发展的良好势头。

　　(三)“三农”工作取得新成效。大力实施强农惠农富农政策。去年，全市财政“三农"支出达到39．95亿元，比上年增长26．6％。大规模铺开农田水利建设。改善灌溉面积19．14万亩，改造中低产田18万亩，新增耕地面积1．13万亩，农业综合生产能力明显增强，全年粮食产量达到19．4亿斤，再创历史新高。加快发展设施农业。新增2．92万亩温室大棚。继续实施规模健康养殖工程。奶牛小区达到215个，奶牛存栏达到14．4万头；肉羊小区达到162个，羊饲养量达到242．8万只；生猪饲养量达到44．5万头。奶牛存栏量、鲜奶产量、人均畜产品占有量、人均牧业纯收入四项指标居全省第一。全力推进“一村一品、一县一业”开发建设。102个村进入省级“一村一品”专业村行列，朔城区被表彰为全国粮食生产先进县，应县被确定为全国蔬菜产业发展重点县，朔城区、怀仁县、山阴县被表彰为全省县域经济发展先进县，右玉县被确定为全省小杂粮基地县。大力推进农业产业化。销售收入超亿元的农产品加工龙头企业达到18家，农产品加工转化率达到41％。扎实推进新农村建设。新增的113个重点推进村全部按要求完成建设任务。加大扶贫开发力度。开展领导干部住村包村和机关单位定点扶贫活动，加大移民搬迁扶贫力度，6400多人实现脱贫。

　　(四)宜居城市建设呈现新气象。中心城市加快发展。实施总投资24亿元的道路、水源、供热、绿化、亮化、净化六大市政基础设施建设工程，城市功能日臻完善。特色城镇建设进一步推进。怀仁县被评为全国文明县城，右玉县被评为中国宜居宜业典范县。造林绿化力度加大。坚持“自己栽树、自己乘凉”的新理念，广泛种植大规格苗木，全市投资12亿元，营造林33万亩，平鲁区被省政府表彰为林业生态县。节能减排成效明显。全市万元地区生产总值综合能耗下降3．5％，万元工业增加值用水量下降14．9％，二氧化硫、化学需氧量、氨氮、烟尘、粉尘等主要污染物减排均完成全年目标任务。市区二级以上天数达到349天，空气质量持续好转。

　　(五)社会事业取得新进步。更加注重保障和改善民生，全市财政在社会事业和民生领域投入达到68．6亿元，占到一般预算支出的59．5％。大力发展教育事业。建设公办标准化幼儿园45所，提高中小学校生均公用经费100元，积极推进中小学校标准化建设，开工建设能源学院。深入推进医药卫生事业改革发展。覆盖城乡的基本医疗保障制度全面建立，全民医保体系初步形成。基层医疗卫生机构普遍实行了基本药物制度，县级医院建设得到全面加强，公立医院改革稳步推进，医疗卫生信息化建设工作走在全省前列，县乡村三级医疗机构达标率达到100％。公共卫生服务水平明显提高，群众“看病难、看病贵”问题得到缓解。积极提升科技创新能力。3家企业被评为省级高新技术企业，25家企业建立了市级技术研发中心。进一步加强文化建设。积极实施文化惠民工程，乡镇综合文化站全部建成。弘扬优秀传统文化，大型北路梆子现代戏《侨女李林》荣获第十五届中国戏剧梅花奖。城乡文化生活丰富多彩，群众性体育活动广泛开展。与此同时，加强人口计生、妇女儿童和残疾人工作，民族、宗教、地震、气象、文物、档案等各项社会事业都取得新成绩。

　　特别是下大力气狠抓了民生工作。稳定和扩大就业。全年新增城镇就业岗位23146个，转移农村劳动力18662人。进一步提高社会保障水平。全市社保基金积累达到30．5亿元。企业养老保险支付能力超过45个月，新型农村养老保险覆盖率达到85％。加大保障性住房建设力度。开工建设各类保障性住房56581套，完成投资50．2亿元，建设规模为历年之最。加快推进农村新的“五个全覆盖”。农村文化体育场所、中等职业教育免费、新型农村社会养老保险三项全覆盖工程两年任务一年完，提前一年完成省定任务。农村街巷硬化和农村便民连锁商店两项全覆盖工程都完成年度目标任务。认真组织实施“暖心煤”工程。免费向农村每户家庭供应一吨煤，保障了农民冬季取暖，全市39．63万户农村家庭受益。

　　全面加强社会管理。坚持不懈狠抓安全生产工作，全年各类安全生产事故起数下降10％，死亡人数下降2．3％。进一步完善应急管理机制。积极推进应急预案体系建设，深入开展应急演练，应急工作得到省军区、省政府应急办和省地震局的好评。突发环境事件应急救援工作得到国家环保部和公安部的肯定，在我市召开全国环境保护与公安消防应急联动机制建设工作交流会。积极支持国防后备力量建设，大力加强人防工作，我市被北京军区表彰为先进人防城市。加强社会治安综合治理和信访工作，社会保持和谐稳定。我市项目化推进社会管理的做法，受到中央综治办的肯定。

　　(六)改革开放增添新动力。全面推进各项改革。行政审批制度改革深入推进，取消、调整行政审批项目108项。事业单位分类改革清理规范工作基本结束。文化体制改革加快推进，我市在全省率先完成了文化体制改革各项任务。金融市场体系得到健全。新增交通银行、招商银行、浦发银行、晋商银行等7家银行分支机构，全市小额贷款公司达到82家。扎实推进转型综改试验工作。市县两级成立了领导组及工作机构，编制完成了全市和试点县行动方案，启动了县区、园区和企业各个层面的试点工作，铺开了一批重大转型标杆项目建设。特别是，经过反复调查研究和科学论证，制定实施了“四化一体”东部新区发展战略，明确了通过工业新型化推动市域城镇化、促进城乡生态化、实现农业现代化的一体化发展的新路子，进一步完善了转型跨越发展思路。去年，东部新区完成地区生产总值725．8亿元，占全市经济总量的84．9％，增长17％，增幅高于全市平均水平1．8个百分点，有力地带动了全市经济社会发展。

　　加大对外开放力度。加强与中煤集团等中央企业的沟通合作，与美国城市规划协会、中国政法大学等国内外学术研究机构签订了战略合作协议。积极参加各类大型招商活动，并组团赴长三角、珠三角等发达地区开展项目推介。全年签约218个招商引资项目，190个落地或开展前期筹备，到位外来资金341．85亿元。

　　(七)民主法制建设取得新进展。自觉接受人大和政协的监督，积极支持各民主党派、工商联、人民团体和无党派人士参政议政。全年共办理人大代表建议和政协委员提案228件。不断推进法制建设，“六五"普法全面启动。完善基层民主制度，第九届村委会换届选举工作顺利完成。深入开展廉政建设，扎实推进煤焦领域反腐败专项斗争。加强干部教育和管理，深入开展纪律作风集中教育整顿月活动，干部作风积极转变，政府自身建设得到加强，政务环境得到优化。

　　各位代表，2011年是我市发展进程中不平凡的一年。在国内外经济环境复杂多变、改革发展任务艰巨的形势下，我市经济加快发展，民生更加改善，在转型跨越发展的道路上迈出了坚实的步伐，开创了科学发展新局面。特别是市第五次党代会进一步明确了工作主题和奋斗目标，完善了发展战略，为“十二五”时期科学发展奠定了思想基础。中央领导和省委、省政府对朔州的工作给予了高度评价。张德江副总理肯定煤炭资源整合煤矿兼并重组“目标非常明确，改革力度很大，效果非常明显”。省委、省政府主要领导肯定我市新的领导班子理念新、眼界宽、标准高，经济发展在多年高位运行的基础上又迈上了新台阶，在全省经济发展中起到了率先和引领作用。

　　过去的一年，成绩来之不易。这是省委、省政府和市委正确领导的结果，是市人大、市政协有力监督和鼎力支持的结果，是全市人民凝心聚力、团结奋斗，辛勤劳动、拼搏进取的结果。在这里，我代表市人民政府，向全市人民和所有关心支持朔州发展的各界人士，表示崇高的敬意和衷心的感谢!

　　在看到成绩的同时，也要清醒地认识到，我市经济社会发展中还存在不少困难和问题。主要是，产业结构不够合理，新兴产业和高新技术产业发展不足；发展方式比较粗放，节能减排和安全生产压力较大，科技和人才对经济社会发展的支撑作用明显不足；农民持续增收难度加大，社会事业发展和民生改善与人民群众的期望还有较大差距；改革开放有待深化，政府职能和干部作风需要进一步转变，发展环境需要进一步优化。对此，我们要高度重视，采取有效措施，认真加以解决。

　　二、2012年工作安排

　　2012年是党的十八大召开之年，是全面贯彻落实市第五次党代会精神、实施“十二五”规划承上启下的重要一年。做好今年的工作，具有十分重要的意义。

　　今年的经济形势严峻复杂，国际金融危机还在发展，世界经济复苏进程艰难曲折；我国经济存在下行压力，经济增速放缓。受国内外影响，能源原材料产品市场需求面临的不确定不稳定因素增加，加之我市经济连续几年高位运行，基数不断扩大，保持经济持续较快增长的难度将越来越大。在看到困难的同时，我们更应看到我国仍处于重要战略机遇期，经济平稳较快发展的大趋势没有改变；我省转型综改试验区建设全面推开，我市被确立为全国农村改革试验区和工业固废综合利用示范基地，我们面临政策叠加的机遇；省委省政府要求我市建设全省转型发展示范区、跨越发展领先区、循环经济集聚区和现代化工业新城，为我们推进转型跨越发展指明了方向；经过多年的持续快速发展，我市经济实力显著增强，发展基础更加坚实；同时，“四化一体”东部新区建设深入推进，平鲁、右玉被列为转型综改试验区“一市两县"试点县，八大工业园区和一批转型标杆项目全面推进，形成了加快发展的强劲态势；特别是，新基地新优势新朔州建设深入人心，极大地激发了全市广大干部群众干事创业的热情，这些都为我们做好今年乃至以后的工作提供了有利条件。我们一定要切实增强机遇意识、创新意识、发展意识，趋利避害，乘势而上，把各项工作做得更好，努力保持经济发展的良好势头。

　　做好今年政府工作，要以邓小平理论和“三个代表”重要思想为指导，深入贯彻落实科学发展观，紧扣“坚定不移推进转型跨越发展、全力打造新基地新优势新朔州"的工作主题，瞄准“建设自然、生态、现代、宜居幸福新城”的宏伟目标，坚持稳中求进的工作总基调，大力推进工业新型化、农业现代化、市域城镇化、城乡生态化，加快转变经济发展方式，在全面推进循环经济上有新成果，在加快发展装备制造业和新兴产业上有新突破，在转型综改先行先试上有新进展，在保障和改善民生上有新举措，在社会管理上有新成效，在加强政府自身建设上有新作为，保持全市经济平稳较快发展和社会和谐稳定，以经济社会发展的优异成绩迎接党的十八大胜利召开。

　　今年经济社会发展的主要预期目标是：地区生产总值完成1000亿元，增长14％；规模以上工业增加值完成560亿元，增长16％；固定资产投资完成593亿元，增长25％；财政总收入完成203亿元，增长16％；一般预算收入完成81．7亿元，增长15．3％；社会消费品零售总额完成192亿元，增长17％；城镇居民人均可支配收入达到22520元，增长11％；农民人均纯收入达到7870元，增长12％；城镇新增就业岗位2万个；居民消费价格涨幅控制在4％左右。

　　约束性指标是：万元地区生产总值综合能耗下降3．5％；二氧化硫、化学需氧量、氨氮排放量分别下降2％、1．3％、1％，氮氧化物排放量实现零增长，烟尘、粉尘排放量均下降3％，万元工业增加值用水量下降3．5％。

　　在实际工作中，我们要把稳增长、控物价、促转型、惠民生、抓改革、保和谐紧密地结合起来。稳增长，充分运用比较优势和有利条件，抓住用好政策机遇，进一步加大项目建设力度，推动全市经济增长速度高于全省平均增速，综合经济实力迈上新台阶。今年全市计划实施省市重点工程178项，总投资2752．5亿元，当年计划完成投资599．1亿元，投资规模超过以往任何一年。控物价，认真贯彻落实宏观调控措施，加强市场监管，保持物价基本稳定，促进消费增长和市场繁荣。促转型，着力推进产业结构调整和发展方式转变，下功夫抓好循环经济、装备制造业和新兴产业，抓好工业园区和转型标杆项目，加快建立资源型产业和非资源型产业均衡发展的机制。惠民生，更加注重保障和改善民生，把促进社会公平正义放在更加突出的位置，切实办成一些让人民群众得实惠的好事实事。抓改革，高举改革开放旗帜，敢于先行先试，勇于率先突破，谋划实施一系列新举措，着力破解发展难题，努力创新体制机制。保和谐，正确处理改革、发展、稳定的关系，积极有效化解各种矛盾和风险隐患，保持社会和谐稳定。

　　今年，重点抓好以下十个方面的工作。

　　(一)强力推进“四化一体”东部新区建设，引领全市转型跨越发展。

　　建设“四化一体”东部新区，是我市贯彻落实省委省政府转型跨越发展战略，紧密结合朔州实际而提出的具有创造性的战略思路。东部新区是我市推进转型跨越发展的重要引擎。今年是东部新区建设实现“两年大变样”的关键一年，区域内各县区、各乡镇要按照东部新区的整体规划加快推进各项工作。要坚持高起点谋划、精细化实施，精心打造以大运公路和北同蒲铁路为轴带，连接中心市区、三个县城、四个重点集镇和六大工业园区，同时建设三条生态绿化带、八大水系的百公里长廊，形成在全省全国具有影响力、在晋北地区具有强大集聚和辐射带动功能的开发示范带。要统筹开发利用地下地上两种资源，做好煤与非煤两篇文章，对矿产资源进行精深加工，对人文历史资源进行深度开发，推动要素集聚、资源集约、产业集群发展，实现工业新型化、农业现代化、市域城镇化、城乡生态化“四化一体”同步推进的目标。要加快启动东部新区核心区建设，举全市之力，努力把东部新区打造成全省转型综改试验区建设的示范区和区域经济发展的新高地。与此同时，切实抓好转型综改试验区“一市两县"平鲁区和右玉县的工作，在全市形成东部新区与西部试点县区竞相发展、互相促进、整体腾飞的良好格局。

　　(二)加快推进工业新型化，努力建设全省循环经济集聚区。 坚持以煤为基、多元发展，紧紧围绕提升资源就地转化率、传统产业循环率、新兴产业占比率、节能减排率、科技贡献率，大力推进工业新型化，加快转变经济发展方式，努力实现单一煤电基地向立体能源基地的转变，实现煤炭资源大市向新兴产业和循环经济强市的跨越。

　　着力改造提升传统产业。煤炭行业，要巩固提升煤炭资源整合煤矿兼并重组成果，进一步规范煤矿建设、生产、经营、安全秩序。加快保留矿井技改步伐，全面推进煤矿标准化建设，年内力争建成22座标准化矿井。支持龙头企业做大做强，推动中煤平朔“双千亿”工程和中煤金海洋“双五百亿”工程加快实施。加强煤炭统一销售管理工作，促进煤炭市场健康发展。电力行业，积极发展高参数大容量电厂，推进煤矸石发电、风电、太阳能发电、生物质能发电等项目，使新能源产业成为我市新的经济增长点。积极推进神头一电厂关小上大2×60万千瓦、华电2×30万千瓦热电联供、大唐新能源利民10万千瓦风电等总装机容量257．3万千瓦的17个续建项目，力争大唐平鲁白玉山20万千瓦风电、晶都太阳能16万千瓦光伏并网电站等项目开工建设。同时，加强电网建设，提高输电能力。乳制品行业，在抓好奶源基地建设的基础上，着力壮大龙头企业，强化新产品研发和市场营销，加快扩大市场份额。陶瓷行业，在巩固提升日用瓷档次的基础上，以市场为导向，优化产品结构，扩大建筑瓷、工艺瓷生产规模，努力打造全国一流的陶瓷基地。要在全市各个行业，组织实施品牌战略，培育一批优秀企业，创立一批名牌产品，提高产业的核心竞争力。

　　大力发展循环经济。坚持把循环经济作为推进工业新型化的基本路径，推动企业、园区、产业循环发展，使循环经济成为我市产业发展的基本模式。以建设全国工业固废综合利用示范基地为抓手，加快构建循环经济技术体系，促进煤基产业链延伸。充分利用资源优势，积极推进煤、电、冶、化一体化发展。坚持把工业园区作为发展循环经济和推进产业转型的主要载体。着力抓好固废综合利用工业园区、朔城区富甲工业园区、平鲁北坪循环经济园区、东露天循环经济园区、山阴北周庄循环经济工业园区、怀仁金沙滩陶瓷工业园区、应县新型产业科技园区、右玉梁威工业园区八大工业园区。每个工业园区都要按照循环经济的理念科学规划，完善设施，加快项目入驻和建设进度。突出抓好固废综合利用工业园区，推动北大固废资源化利用产学研示范基地等项目加快建设，为粉煤灰高效利用发挥示范作用。八大工业园区是我市重要的经济增长极，要精心打造，全力支持，使之成为转型跨越发展的标志性工程。经济开发区要以扩区为契机，坚持项目立区、招商兴区、机制活区，科学规划功能区域，切实加强基础设施建设，全面创新管理方式，全力推进招商引资，加快引进建设一批标志性重点项目，尽快走上科学健康发展轨道。

　　加快发展装备制造和新兴产业。装备制造业薄弱，新兴产业缺乏，是我市经济结构中的致命弱点。实现转型发展，必须把加快发展装备制造业和新兴产业作为主攻方向和着力点。要下力气加快发展具有一定基础和比较优势的煤机装备制造、煤化工、新型材料、特色食品、生物医药产业，发展科技含量高、成长潜力大的节能环保、信息技术等高新技术产业，力求在不太长的时期内取得较大突破。探索建立促进装备制造业和新兴产业发展的激励机制。各级政府要加大对转型标杆项目的扶持力度。鼓励引导从煤炭领域退出的民间资本投入转型领域，实现二次创业，回报社会，回报家乡。今年，重点抓好中煤平朔120万条子午线轮胎、城区大唐金凤风机组装、怀仁天磊年产10万吨涂布级高岭土、应县赛特电梯、应县10万吨高效减水剂、右玉光电绿色科技产业园、雨润集团怀仁和右玉肉食品加工等项目。

　　各位代表，转型发展任重道远，前景广阔。只要我们坚持发展不松劲，狠抓转型不动摇，调整结构不懈怠，我们就一定能够推动我市的产业结构发生脱胎换骨的新变化！

　　(三)加快推进农业现代化，当好雁门关生态畜牧经济区建设排头兵。

　　“三农”工作始终是各项工作的重中之重。在工业化和城镇化加快推进的进程中，要更加重视农业现代化。

　　进一步加大强农惠农富农政策支持力度。今年市本级财政将在“三农”领域投入4．1亿元，加大农业生产补贴力度，加强农业农村基础设施建设。新增农业补贴重点向种养大户、农民专业合作社及各种生产服务组织倾斜。

　　切实加强农田水利建设。扎实抓好应县、山阴、怀仁三个全国小型水利重点县建设，积极推进大中型灌区节水改造和农业节水示范工程，新增高标准节水灌溉面积6万亩。开工建设神头泉水源保护、七里河市区段综合整治、西山引黄灌区、桑干河灌区扩建和恢河调蓄水整治五项重点水利工程，加快提升水利建设水平。加快改造中低产田，加大盐碱地综合治理力度，年内力争改良10万亩盐碱地。

　　大力发展特色现代农业。我市位于奶牛、肉羊、小杂粮的黄金产区，发展特色农业优势明显，我们一定要做好特色农业这篇大文章。要集中培育生态畜牧和杂粮两大特色品牌，把雁门关生态畜牧经济区打造成大品牌，把小杂粮做成大产业。加快发展高效设施农业。新增设施农业4万亩。重点抓好园区建设。东部新区4个县10个万亩园区要加大建设力度，年内形成雏形。红旗牧场现代农业高新技术示范园区和平鲁下水头万亩设施农业园区要完成规划，开工建设。大力推进规模健康养殖。新建奶牛标准化养殖小区30个、肉羊标准化养殖小区50个。实施振兴奶业苜蓿发展行动，打造6个苜蓿种植专业示范村。大力发展循环农业，加速农业向高产、优质、高效发展。扎实推进“一村一品、一县一业”。朔城区重点发展城郊型农业，平鲁区重点发展马铃薯产业，山阴县重点发展奶牛产业，应县重点发展蔬菜产业，怀仁县重点发展养羊产业，右玉县重点发展小杂粮产业。在抓好102个省级专业村的基础上，新增120个专业村。加快提高农业产业化经营水平。积极扶持进入全省“513”工程的古城、天鹏等龙头加工企业做大做强，推广“公司+专业合作社+基地+农户”的产业化经营模式。着力抓好特色农产品精细加工，努力打造一批在全省全国叫得响的特色农产品品牌。精心筹办首届雁门关生态畜牧经济区特色农产品博览会。同时，瞄准京津等周边城市，积极开展特色农产品推介活动，加强农超对接，进一步提高我市特色农产品知名度。

　　加快推进农业科技进步。大力推动农业科技创新，加大对良种繁育、疾病防控、农产品质量安全等关键技术研发和推广应用的支持力度。加强农业科技人才队伍建设，切实增强基层农技推广服务能力，积极开展农村实用人才培训。推进12316农技咨询热线和远程教育网络建设，促进先进适用技术到田到户。加快农业机械化步伐，加大农机新技术和新机具推广力度。

　　深入推进新农村建设。新增185个重点村，全市新农村重点村达到818个，占到全市农村总数的一半。要以全面完成农村新的“五个全覆盖”任务为重点，提升新农村建设水平。各县区要足额配套建设资金，确保农村新的“五个全覆盖"工程保质保量圆满完成。深化农村改革，开展土地承包经营权确权颁证工作，积极稳妥推进土地流转，鼓励发展适度规模经营。

　　进一步加大扶贫开发力度。认真实施国家新十年扶贫开发纲要，按照新的扶贫标准，不断加大投入和工作力度，让更多低收入农民的生活得到保障。结合农村危房改造、地质灾害治理、生态移民，做好移民扶贫搬迁。逐步将300人以下、实际居住人口不到一半的偏远山区贫困村向中心村搬迁。今年要移民扶贫4000人。继续实施整村推进项目。今年全市要完成53个贫困村整村推进任务。健全“一企一村、一矿一村”扶贫机制，推动产业扶贫。

　　各位代表，农民收入翻番是“十二五”时期的一项重要任务。我们要紧紧围绕这一目标，不断加大“三农”工作力度，努力实现农业强、农民富、农村美，让农民兄弟过上更加幸福美好的生活!

　　(四)加快推进市域城镇化，建设城乡经济社会一体化发展先行区。

　　要以建设全国农村改革试验区为平台，以城乡经济社会发展一体化制度建设为抓手，加快推进城镇化进程，促进城乡一体化发展，形成城镇化与工业化、城镇化与新农村建设良性互动的发展格局。 科学规划城乡空间布局。要按照全省“一核一圈三群”的总体城镇布局，坚持中心城市、大县城、小集镇、新农村“四位一体”发展战略，统筹规划城镇建设、农田保护、产业集聚、村落分布、生态涵养、文化传承，全市城乡空间要形成布局科学合理、功能配套完善、居住相对集中的发展格局。突出规划引领作用，今年要全面完成市县土地整治规划编制、县乡土地利用总体规划编制、城市总体规划修编、市区50平方公里核心区域控制性详细规划编制，调整优化城乡空间布局。

　　充分发挥中心城市的龙头作用。突出城市让人民生活更美好的主题，按照“提升中心区、改造旧城区、拓展新市区、充实开发区、建设生态区”的总体部署，加大道路、水源、供热、绿化、亮化、净化基础设施建设力度，进一步拉开城市框架，提高城市综合承载能力，提升城市品位。加快推进中心区的品质提升，彰显城市特色品格。加快推进旧城区改造，使古城焕发出新的生机。加快打造新市区，不断完善城市服务功能。加快充实开发区，形成城市发展新的增长极。加快推进生态区建设，打造城市绿色生态屏障。要科学规划城市功能区域，推进城市协调有序发展。今年要抓好恢河大桥、朔州西互通连接线、振武街东拓、滨河路打通、平朔南路打通、建设北路及市府东街改造、七里河公园、绿化工程、热电联产集中供热、北旺庄和下庄头城中村改造等十大重点工程，提高城市的通达性、便捷性和舒适度。要积极稳妥地推进城中村改造，做到依法拆迁、和谐拆迁。以创建国家园林城市为目标，在市区创建3个星级公园、8条园林式道路、60个园林小区。要理顺城市建设管理体制。认真落实《城乡规划法》，进一步明确市区、城区、开发区在城市规划、建设、管理方面的权限，形成统一规划、市区衔接、部门协调、权责对应、执法有力、规范严格的城市管理体制。大力实施城乡环境综合整治，着力解决环境卫生、市场管理、交通秩序等方面存在的突出问题，改善城乡环境面貌。

　　强化县城和中心集镇的集聚带动作用。积极实施“大县城”战略，加快县城扩容提质步伐，努力打造各具特色的工业强镇、商贸重镇和旅游文化名镇。平鲁区要继续推进“一城十镇百村”整体规划。山阴县要按照“一年一个样、三年大变样”的要求，改旧城、建新城，加快改变县城面貌。怀仁县要巩固提升城市建设水平，创新管理模式，建设华北最美县城。应县要依托木塔，打造历史文化名城。右玉县要加强生态建设，开发旅游产品，打造塞外旅游胜地。县城和集镇要充分发挥其连结城乡、集聚产业的优势，转移吸纳周边农村人口，切实成为城镇化建设的重要支点。

　　加快交通网络建设。配合好北同蒲增四线工程、大西高速客运专线、准朔铁路、大准至朔黄连接线、准朔铁路朔山连接线等铁路工程建设，继续推进高速公路、干线公路、农村公路建设，完善综合交通体系。今年要建成平朔高速公路，实现县县通高速，推进荣乌高速山阴至平鲁段建设，力争西纵高速公路朔州段开工建设，加快构建完整的高速公路网。完成大呼高速公路右玉连接线建设任务，推进虎山线山阴段改造工程，开工建设大石线应县县城段改线、109国道右玉县城段改线、大忻线山阴县城段西移改线和平万线虎头山隧道，推进干线公路等级化。开工建设朔神大道东延线，连通市区和固废工业园区，为东部新区核心区建设创造良好的交通条件。开工建设朔广线至紫金山旅游公路和环广武旅游公路，促进旅游业发展。全面完成农村街巷硬化全覆盖工程和康家窑至马营堡农村公路改造工程(神电运煤通道二期工程)，改造朔城区至山阴县、应县经济开发通道，在村村通油路的基础上，进一步推进农村路网建设。

　　着力破除农民融入城市的体制机制障碍。推进户籍制度改革，放宽城镇落户条件，有序将在城镇稳定就业和居住的农民工转变为城镇居民。加强对农民工的人文关怀和服务，逐步将社会保障、就业服务、医疗卫生、文化教育和保障性住房等城镇基本公共服务覆盖到农民工，让农民无论进城还是留乡，都能安居乐业、幸福生活。

　　朔州是一座年轻而充满希望的城市，发展空间广阔，发展潜力无限。只要我们人人热爱朔州，齐心协力建设朔州，我们一定能够用勤劳智慧的双手，把朔州建设得更加美好，不断谱写幸福新城建设的崭新篇章!

　　(五)加快推进城乡生态化，建设自然生态绿色朔州。

　　大力推进造林绿化。要科学规划布局，坚持生态效益、社会效益和经济效益统筹兼顾，山上治本、身边增绿和生态富民同步推进，进一步加大植树造林力度。深化集体林权制度改革，健全公益林补偿机制。优化树种结构，实现经济林与生态林协调发展。加强林木管护，严防森林火灾发生。今年继续完成营造林33万亩。重点抓好八大规模造林工程，即朔城区西山环城绿化六期工程5万亩、平鲁区环城绿化工程4万亩、山阴县西山和广武拓展工程4万亩、怀仁县金沙滩生态经济园林区三期工程6万亩、应县荣乌高速公路通道绿化工程44公里、右玉县大呼高速连接线沿线荒山绿化4万亩、大运高速公路盐碱地绿化示范工程12公里(平川四县区每个县区3公里)、大运公路沿线绿化完善工程100公里。鼓励社会力量投资建设苗木繁育基地。每个县都要规划建设一座万亩苗圃。

　　加强矿山生态环境修复治理。严格执行生态环境恢复治理保证金制度，完善生态环境修复治理长效机制。按照“谁破坏、谁修复”和“谁投资、谁受益”的原则，推行“一矿一企治理一山一沟”，加大矿山生态修复治理力度。抓好矸石山治理、采空沉陷区治理、矿井水净化利用以及地下水资源保护，推行绿色开采，建设生态矿山。

　　强力推进节能减排。加大落后产能淘汰力度，从严控制“两高一资”项目，支持重点节能项目建设。以创建省级环保模范城市为目标，强化污染减排措施。加强重点排污企业的监管和治理，抓好电厂燃煤机组脱硫脱硝，关停天诚电冶等污染企业。抓好农业源污染减排、机动车污染减排，推进市区垃圾处理厂、医疗废物处理厂、污水处理厂中水回用等环保工程建设，进一步削减排污总量，改善市区大气污染状况。

　　各位代表，改善生态环境，建设资源节约型、环境友好型社会，是全市人民的热切期盼。我们一定要大力发扬右玉精神，坚持不懈植树，狠下决心治污，让朔州大地山更绿、水更清、天更蓝、空气更清新，让我们的家园更加美丽!

　　(六)加快发展服务业，促进产业结构调整。

　　服务业是我市经济发展中的短板。加快发展服务业是产业结构调整的根本出路，是城镇化的必然要求。必须下大力气改变我市服务业发展缓慢、比重偏低的现状。在做优第一产业、做强第二产业的同时，加快做大第三产业。把服务业作为稳增长、调结构、促就业、保民生的重要支撑，坚持生产性服务业与生活性服务业并重，传统服务业与现代服务业并举，促进服务业发展提速、比重提高、水平提升。

　　统筹发展各类服务业。加快发展大型物流、机电维修等生产性服务业，促进服务业与支柱产业良性互动。鼓励发展房地产物业服务、社区服务、家政服务和社会化养老服务等生活服务业，方便群众生活。升级改造公共交通、宾馆餐饮、文化娱乐、商贸流通业等传统服务业，完善城市服务功能。积极发展信息、金融、会展、电子商务、外包服务等现代服务业，引领经济发展。大力发展总部经济，提高城市的集聚辐射力。规划建设大型商贸综合体，着力打造新型商务区和特色商业街，提升城市形象。新建改造一批标准化菜市场，切实解决马路市场和农民卖菜难问题。巩固“万村千乡市场工程”成果，继续推进“家电下乡"、“汽车下乡”，扩大农村消费市场。重点抓好朔城区企业总部基地、准池铁路物流园区、平朔物流园区、居然之家仓储中心、怀仁华北皮革城、山阴县雁门关农副产品物流园区、朔州宾馆、戴思宾馆、金沙国际大酒店等项目。

　　重点发展旅游业。以旅游规划为先导，以景区建设为支撑，大力整合各类旅游资源，深入挖掘文化内涵，全面提升旅游业品质，努力将朔州打造成北方特色旅游目的地。以应县木塔申报世界文化遗产为龙头，加快广武边塞文化旅游区、崇福寺、紫金山、金沙滩、平鲁门神文化园和全景右玉生态文化旅游区等重要景区的开发提升步伐，打造精品线路，健全旅游服务体系，加大旅游宣传促销力度，拓展旅游市场，促进旅游业与文化产业、城市建设融合发展。

　　加大政策支持力度。制定实施有利于服务业发展的政策，支持社会资本进入服务业领域。对鼓励类服务业，实行与工业用电、用水、用气、用热同价政策，在土地、金融、财税等方面给予倾斜。加大政府资金扶持力度，支持带动力强的服务业示范项目。

　　(七)深入实施科教兴市和文化强市战略，推动社会事业加快发展。

　　坚持优先发展教育。认真贯彻落实国家和我省中长期教育规划发展纲要，今年全市教育支出要达到一般预算支出的17．02％。加快发展学前教育，每个乡镇都要建设好一所中心幼儿园。推进义务教育学校标准化建设，加强农村寄宿学校建设，优化城市学校布局，切实解决上学难和班容量大的问题。积极发展职业教育，每个县都要办好一所高标准的职业高中。大力发展高等教育。加快提升职业技术学院办学水平，为农业现代化建设培养更多的紧缺实用人才。进一步完善朔州师专配套设施，争取年内通过教育部验收。加快建设能源学院，积极推进怀仁陶瓷学院建设，为支柱产业提供有力的人才支撑。

　　做好科技和人才工作。加大科技投入，完善科技创新和服务体系。在循环经济、特色农畜产品良种培育、新材料、生态修复等转型发展的重要领域实施重大专项攻关。规划建设科技创新园区．，促进高新技术产业集聚发展。支持企业加强研发中心建设，实现产学研有机结合，加快科技成果转化。依托北大产学研基地等项目，建设博士后工作站。全面加强人才工作，加大人才培养引进力度。“十二五”期间，市政府将每年投入500万元，用于干部培训，提高干部素质。实施“3个100”科技人才引进计划，全市聘请100名专家，联系100所高校，引进100名紧缺人才。人才是最稀缺最宝贵的战略资源。各级各部门和全社会都要关心人才、爱护人才，使朔州真正成为人才集聚、活力迸发的人才高地!

　　加快发展医疗卫生事业。深化医药卫生体制改革，巩固扩大基本医疗覆盖面，提高基本医疗保障水平和管理服务水平。城镇居民医保和新农合补助标准提高到每人每年240元。巩固完善基本药物制度，全面推进公立医院改革，加强基层医疗卫生服务体系建设。加快实现计划生育优质服务全覆盖，将免费孕前优生健康检查范围扩大到80％。加强流动人口计划生育服务管理。

　　推动文化大发展大繁荣。深入推进社会主义核心价值体系建设，用科学发展观理论教育干部群众。加强社会公德、职业道德、家庭美德和个人品德教育，动员社会各界广泛开展群众性精神文明创建活动，提升市民素质，提高全社会文明水平，争创全国文明城市。进一步提炼城市精神，提升城市文化软实力，增强城市凝聚力。健全公共文化服务体系。大力实施文化惠民工程，市县(区)要加快建设一批标志性文化设施。完善新闻大楼、博物馆、图书馆等重点文化工程，尽快投入使用。加强文物和非物质文化遗产保护。加快发展文化产业，促进文化创意、演艺娱乐、文化旅游等产业加快发展。深化文化体制改革，巩固经营性文化单位改革成果，推进公益性文化事业单位内部改革。认真贯彻落实朔州市《全民健身实施计划(2011—2015年)》，大力开展群众健身活动，加强体育基础设施建设。

　　(八)着力保障和改善民生，让人民群众享受更多的改革发展成果。

　　稳定和扩大就业。实施更加积极的就业政策，鼓励发展劳动密集型企业，多渠道开发就业岗位，加大公益性岗位开发力度，全面做好各类人员的就业工作，重点做好高校毕业生、农村转移劳动力和城镇下岗职工培训就业工作。要高度重视下岗职工再就业工作。推进创业就业工程，落实创业扶持政策，以创业带动就业，形成全民创业氛围。全面实行劳动合同和备案制度，加强企业特别是煤炭企业和建筑施工企业的劳动用工管理。加大农民工权益保护力度，实施农民工工资保证金制度，确保农民工按时足额领到工资。

　　完善社会保障体系。进一步扩大社会保障覆盖面，提高统筹层次和保障水平。积极发展老龄事业，加快建设社会养老服务体系，推进城镇居民社会养老保险和新型农村社会养老保险工作，确保城乡居民养老保险实现全覆盖，努力让城乡每个老年人都老有所养，幸福安度晚年。关心低收入群众生活，完善城乡低保管理工作，逐步扩大低保覆盖面，做到应保尽保。城乡低保标准每人每月分别提高30元、22元。进一步提高农村五保户供养标准。加强社保服务能力建设，推进社会保障卡发放。积极发展社会福利和慈善事业，完善城乡社会救助体系。

　　加快保障性住房建设。新建各类保障性住房34352套，完成投资45．956亿元。制定完善保障性住房建设、分配、管理制度，确保公平分配，切实保障低收入困难家庭的基本住房需求。同时，合理规划商品房开发，促进房地产市场健康发展。

　　加强和创新社会管理。毫不松懈地抓紧抓好安全生产工作。始终坚持“安全第一”的理念，时刻绷紧安全生产这根弦，警钟长鸣，常抓不懈。要以深入贯彻落实国务院和省政府关于加强安全生产工作的政策法规为主线，以落实企业安全生产主体责任和政府安全监管主体责任为保障，以深化安全生产专项整治为手段，以事故预防为主攻方向，继续开展“安全生产年”活动，推进安全生产标准化建设，进一步抓好煤矿、非煤矿山、交通运输、食品药品、消防、校园等各行业各领域的安全工作，坚决杜绝各类重特大事故，促进全市安全生产形势持续稳定好转。各级各部门和企业要把安全生产作为重要考核内容，严格实行“一票否决制”，严肃事故责任追究，保证各项安全生产措施落到实处。加强社区建设，提高基层社会管理服务效能。完善信访工作和矛盾调处机制，正确处理各类社会矛盾，切实解决好土地征用、房屋拆迁、劳资纠纷等事关人民群众切身利益的问题。进一步加强应急管理，健全突发事件应急体系，做好防灾减灾工作。进一步完善社会治安防控体系，严厉打击各类违法犯罪活动，维护社会和谐稳定。发展人民防空事业，支持国防后备力量建设，积极开展省级双拥模范城市创建活动，促进军民融合式发展。

　　各位代表，人民群众是我们的衣食父母。怀着对群众的深厚感情，着眼于解决广大群众最关心、最直接、最现实的利益问题，今年市政府承诺为民办好八件实事。一是实施义务教育阶段寄宿学生饮用奶工程。二是为全市所有义务教育阶段学生免除教科书费用。三是开展送戏下乡活动。四是实施爱心助残工程。五是新建市社会福利院、儿童福利院。六是建设朔州市劳动力市场。七是建设朔州市创业孵化基地。八是为市区70岁以上老年人、残疾人、伤残军人发放公共交通补贴。

　　群众利益无小事。我们一定扎扎实实、高标准、高质量办好每件实事，让群众满意。我们将坚持每年办成几件群众关心的好事实事，解决群众生产生活中的难点热点问题，让广大人民群众不断享受到越来越多的改革发展成果，生活得越来越幸福!

　　(九)进一步深化改革开放，努力构建适应科学发展的体制机制。

　　朔州是伴随着改革开放成长起来的，没有改革开放，就没有朔州。在推进转型跨越发展的新时期，我们要继续发扬敢为人先、勇于作为、与时俱进的精神，奋力开创改革开放新局面。

　　继续深化各项改革。扎实推进扩权强县试点工作，进一步扩大县域发展的自主权。积极推进事业单位改革，全面推行事业单位人员公开招聘制度。继续推进国有企业和集体企业改革，积极扶持民营企业，支持引导非公有制经济发展。积极创新用地保障机制，认真落实露天采矿用地改革、矿业存量土地整合利用、城乡建设用地增减挂钩和工矿废弃地复垦调整利用等试点政策，探索将集中连片重度盐碱地开发为工业用地。通过多种渠道，努力为城镇化和工业化提供有力的土地支撑。大力推进财政金融创新。千方百计增加财政收入，科学合理安排财政支出，多方筹集建设资金，有效服务转型跨越发展。加快发展资本市场，积极支持企业上市和发行债券，力争在两年之内企业上市实现零突破。进一步推进银企合作，努力扩大信贷规模。加强地方融资平台建设，形成财政、金融与重点项目建设良性互动机制。发展壮大担保机构，扩充担保机构资本金，为中小企业融资创造宽松环境。鼓励民间资本进入担保行业。

　　继续扩大对外开放。进一步优化发展环境，提升服务质量，加大招商引资力度，吸引更多的国内外大企业投资落户。积极开展“项目落地年”活动，抓好中博会等招商活动签约项目的落地工作，提高项目落地率。搞好跟踪服务，积极争取正大集团综合性现代农业示范项目、罗克佳华工业公司“智慧平鲁”等项目早日启动建设。精心组织各项重点招商活动，深化与中煤集团、中电国际、华电集团、神华集团等大企业的合作。今年力争落地招商引资项目76个，到位外来资金427亿元。

　　全面推进转型综改试验工作。资源型地区转型是加快转变经济发展方式的战略选择，是科学发展观的必然要求。我市资源型经济正处于成长期，早转型早主动。山西省国家资源型经济转型综改试验区的启动建设，为我们提供了大机遇、大平台、大载体。我们一定要紧紧抓住这个难得的历史机遇，以转型综改试验工作为抓手，全面深化改革开放，着力破解发展难题，为朔州的长远发展赢得主动、赢得优势、赢得未来。要按照省政府批复的行动方案，加快推进“一市两县”、“一市两园”和“一县一企”等试点工作，大力推进重大转型标杆项目建设，在产业升级、生态修复、城乡统筹、民生改善等重点领域和体制机制创新上先行先试、取得突破，努力在全省率先走出一条资源型地区转型跨越发展的新路子。

　　(十)加强政府自身建设，为转型跨越发展创造良好的政务环境。

　　良好的环境是制胜的砝码。要按照科学执政、民主执政、依法执政的要求，以建设服务、责任、法治、廉洁政府为目标，积极加强政府自身建设，不断提高政府工作水平，为转型跨越发展创造良好的政务环境。

　　规范政府行为，推进依法行政。各级政府要严格依照法定权限和程序行使权力，履行职责。自觉接受人大及其常委会监督，主动加强与政协联系，认真办理各类建议和提案。坚持科学决策、民主决策，重大决策都要广泛征求意见，进行严格论证。坚持依法治市，加强法制宣传教育，深入推进“六五”普法工作。严格依法行政，深化行政执法监督，加强行政监察和审计监督，不断增强政府的公信力和执行力。

　　提高行政效能，优化政务服务环境。积极推进服务型政府建设，不断提高公共服务水平。深入推进行政审批制度改革，精简审批事项，规范行政许可。具有行政审批职能的市政府部门，要按照职能归并原则，设立行政审批服务办公室。大力推进政务公开，加强政务服务中心建设，集中办理行政审批事项和服务事项，要建立重大项目审批和服务企业的绿色通道，建立规范的公共资源交易平台，让权力在阳光下运行。

　　坚持为民务实，加强作风建设。广大公务员尤其是各级领导干部要大力弘扬右玉精神，坚持求真务实，真抓实干；要经常深入基层、深入群众，问计于民、问政于民、问需于民；要深入调查研究，发现问题，分析问题，解决问题，推动工作落实。进一步完善领导干部住村包村、联系企业和机关帮扶基层工作机制。市直部门要认真开展“五个一”活动，即聘请一名专家，引进一个项目，研究出台一项先行先试的政策，推出一项提高行政效能的措施，联系帮扶一个基层单位，以实际行动推动转型跨越发展。要加强绩效管理，强化督查考核，确保改革发展各项工作部署落到实处、收到实效。

　　坚持反腐倡廉，加强廉政建设。各级政府及领导干部要认真落实党风廉政建设责任制，加强廉政风险防控，健全惩治和预防腐败体系。各级领导干部要坚持为民务实清廉，认真遵守《廉政准则》，严格执行“一岗双责”，当好勤政廉政表率。继续深入开展煤焦领域反腐败工作，认真开展重点领域渎职侵权问题专项整治，严肃查处各类违法违纪案件。持续开展纪律作风整顿，严明工作纪律，狠刹懒政怠政、不负责任等不良风气，坚决查处吃拿卡要、以权谋私的典型案例，努力营造风清气正、干事创业的良好氛围。

　　各位代表，朔州是一个出生产力、出战斗力的地方，是一片充满无限生机活力的沃土。这片沃土赋予我们使命，给予我们激情，更使我们增添了干事创业的信心和决心。当前，朔州已经进入转型跨越发展的新时期。站在新的历史起点，我们要以新的精神风貌、新的更大作为，创造新的辉煌业绩，不负时代重任，不负人民期望。让我们在中共朔州市委的坚强领导下，解放思想，开拓创新，扎实工作，不断开创转型跨越发展的新局面，为打造新基地新优势新朔州、建设自然生态现代宜居幸福新城而努力奋斗!
